# Impact of emergency intubation on central venous oxygen saturation in critically ill patients: a multicenter observational study

**DOI:** 10.1186/cc7802

**Published:** 2009-05-04

**Authors:** Glenn Hernandez, Hector Peña, Rodrigo Cornejo, Maximiliano Rovegno, Jaime Retamal, Jose Luis Navarro, Ignacio Aranguiz, Ricardo Castro, Alejandro Bruhn

**Affiliations:** 1 Pontificia Universidad Católica de Chile, Departamento de Medicina Intensiva, Marcoleta 367, Santiago, Chile; 2 Instituto Nacional de Cardiología Ignacio Chávez, UTI de Cardio-Neumología, Juan Badiano No. 1 C.P. 14080, Ciudad de México, México; 3 Hospital Clínico Universidad de Chile, Unidad de Pacientes Críticos, Santos Dumont 999, Santiago, Chile

## Abstract

**Introduction:**

Central venous oxygen saturation (ScvO_2_) has emerged as an important resuscitation goal for critically ill patients. Nevertheless, growing concerns about its limitations as a perfusion parameter have been expressed recently, including the uncommon finding of low ScvO_2 _values in patients in the intensive care unit (ICU). Emergency intubation may induce strong and eventually divergent effects on the physiologic determinants of oxygen transport (DO_2_) and oxygen consumption (VO_2_) and, thus, on ScvO_2_. Therefore, we conducted a study to determine the impact of emergency intubation on ScvO_2_.

**Methods:**

In this prospective multicenter observational study, we included 103 septic and non-septic patients with a central venous catheter in place and in whom emergency intubation was required. A common intubation protocol was used and we evaluated several parameters including ScvO_2 _before and 15 minutes after emergency intubation. Statistical analysis included chi-square test and t test.

**Results:**

ScvO_2 _increased from 61.8 ± 12.6% to 68.9 ± 12.2%, with no difference between septic and non-septic patients. ScvO_2 _increased in 84 patients (81.6%) without correlation to changes in arterial oxygen saturation (SaO_2_). Seventy eight (75.7%) patients were intubated with ScvO_2 _less than 70% and 21 (26.9%) normalized the parameter after the intervention. Only patients with pre-intubation ScvO_2 _more than 70% failed to increase the parameter after intubation.

**Conclusions:**

ScvO_2 _increases significantly in response to emergency intubation in the majority of septic and non-septic patients. When interpreting ScvO_2 _during early resuscitation, it is crucial to consider whether the patient has been recently intubated or is spontaneously breathing.

## Introduction

Central venous oxygen saturation (ScvO_2_), a complex physiologic parameter, is being widely used as a resuscitation goal in critically ill patients [1-3], although several limitations may preclude a clear interpretation of its changes [4]. Early therapeutic interventions applied rather simultaneously after hospital or intensive care unit (ICU) admission, may affect the oxygen transport (DO_2_)/oxygen consumption (VO_2_) balance and ScvO_2 _in an unpredictable direction. The uncommon finding of low ScvO_2 _values in critically ill ICU patients may be explained by the predominately positive impact of these early interventions [5,6].

More than 70% of critically ill patients undergo emergency intubation during ICU stay [6-8], a maneuver with strong and eventually divergent effects on the physiologic determinants of DO_2 _and VO_2_. The final impact of emergency intubation on ScvO_2 _may be unpredictable since it could potentially increase ScvO_2 _by blunting regional VO_2_, or eventually decrease it, particularly in hemodynamically unstable or hypovolemic patients, due to the negative effects of sedation and positive intra-thoracic pressure on cardiac output. Of note, 53% of septic patients were intubated during the study period in the early-goal directed therapy (EGDT) trial [1], but the impact of this intervention on ScvO_2 _was not reported, nor has it been studied thereafter.

Our aim was to study the specific impact of this isolated maneuver on ScvO_2 _in critically ill septic and non-septic patients subjected to emergency intubation.

## Materials and methods

This prospective observational multicenter study was performed in three university-affiliated hospitals between December 2006 and March 2008. The study was approved by the corresponding institutional review boards. Surrogates signed an informed consent for ICU treatment including the intubation procedure.

### Inclusion and exclusion criteria

Adult patients with arterial and central venous catheters in place with a confirmed tip position in the superior vena cava, and in whom emergency intubation was required, were enrolled. Patients with acute neurological conditions and post-cardiac arrest were excluded.

### Study protocol

The intubation protocol started as soon as the intubation was decided. It included pre-oxygenation with 100% oxygen, etomidate (0.1 to 0.3 mg/kg) or propofol (0.5 to 2 mg/kg) for unconsciousness induction. Fentanyl (1 to 5 μg/kg), midazolam (0.01 to 0.1 mg/kg), and rocuronium (0.6 to 1.2 mg/kg) were used for sedation and neuromuscular paralysis. Mechanical ventilation was started in all patients with the following initial settings: fraction of inspired oxygen (FiO_2_) 100%, respiratory rate (RR) 15 breaths/minute, tidal volume of 8 ml/kg and positive end expiratory pressure (PEEP) 5 cmH_2_O. If hypotension developed during intubation, a bolus of 250 ml of saline solution was infused and vasopressors were administered as required.

The study period was 15 minutes. Arterial and central venous samples were drawn for blood gases analysis immediately before and 15 minutes after intubation. Simultaneously, the following clinical variables were recorded: arterial pressure, heart rate (HR), and RR. After the second blood gas samples, ventilator parameters were adjusted according to the particular patients requirements and current recommendations [2]. Blood samples were placed in ice cold water and transferred to the central laboratory to be analyzed by co-oximetry (ABL 725; Radiometer, Copenhagen, Denmark). Oxygen extraction ratio (O_2_ER) was calculated as O_2_ER = 100 × (SaO_2 _- ScvO_2_)/SaO_2_, where SaO_2 _is arterial oxygen saturation.

The clinical characteristics of the patients, demographic variables, cause of intubation, use of vasoactive drugs, and severity scores (Acute Physiology and Chronic Health Evaluation (APACHE) II and Sequential Organ Failure Assessment (SOFA)) were recorded at baseline. After the emergency, patients were classified as septic or non-septic, according to the predominant condition that led to the cardio-respiratory failure. Changes in ScvO_2 _were analyzed for the whole population and also individually for septic and non-septic subgroups.

### Statistical analysis

Numerical variables were compared using Student's t test, and categorical variables were compared by chi-square or Fisher's exact test. Changes in variables (ScvO_2_, O_2_ER) were analyzed by a paired Student's t test. Correlation between changes in ScvO_2 _and SaO_2 _was performed with linear regression analysis. The SPSS 17.0 software (Chicago, IL, USA) was used for statistical calculations. Results are expressed as percentages or mean (± standard deviation). A *P *< 0.05 was considered as statistically significant. All reported *P *values are two-sided.

## Results

A total of 108 critically ill patients requiring emergency intubation were included in this study. Forty-two patients (40.8%) were intubated for respiratory failure, 17 (16.5%) for circulatory failure, and the remaining 44 (42.7%) for mixed causes. Five patients were excluded from analysis because measurements could not be obtained in due time: two with difficult intubation and three for severe cardiovascular instability during the procedure. In these patients, samples were taken only after 35 to 50 minutes, and ScvO_2 _ranged from 59 to 65% with no improvement compared with pre-intubation values.

Baseline characteristics of the remaining 103 patients are shown in Table 1. Forty-eight patients (46.6%) had severe sepsis (more frequently respiratory (43%) and abdominal (40%) sources). These patients had septic shock, community-acquired pneumonia, pancreatitis, and postoperative sepsis, with different organ dysfunction profiles including acute lung injury (ALI)/acute respiratory distress syndrome (ARDS) in 20 (42%). Fifty (91%) of the non-septic patients were of cardiogenic origin (including acute circulatory failure, acute coronary syndromes, pulmonary edema, pulmonary thromboembolism, life-threatening arrhythmias, and congestive heart failure).

**Table 1:** Baseline characteristics of the patients

	All patients (n = 103)
Age (years)	58 ± 17
Gender male/female, n/(%)	65 (63.1)/38 (36.9)
APACHE II score	26 ± 7
SOFA score	9 ± 4
Hemoglobin (g/dl)	10.3 ± 1.9
Presence of severe sepsis	
Yes, n (%)	48 (46.6)
No, n (%)	55 (53.4)
Cardiogenic, n (%)	50 (48.5)
Vasoactive drug use	
None, n (%)	41 (40)
Vasopressors, n (%)	33 (32)
Norepinephrine	20 (19)
Dopamine	5 (5)
Inotropes, n (%)	29 (28)
Dobutamine	17 (17)
Milrinone	5 (5)
Levosimendan	2 (2)
Vasoactive dose	
Norepinephrine, μg/kg/min	0.1 ± 0.1
Dopamine, μg/kg/min	5.2 ± 2.6
Dobutamine, μg/kg/min	4.6 ± 1.9
Milrinone, μg/kg/min	0.42 ± 0.21
Levosimendan, μg/kg/min	0.2 ± 0.1

APACHE = Acute Physiology and Chronic Health Evaluation; SOFA = Sequential Organ Failure Assessment.

At intubation, 41 patients were macro-hemodynamically stable without vasoactive drugs, and the others used either vasopressors or inotropes as shown in Table 1. Basal arterial lactate was 2.27 ± 1.77 mmol/L. Severe septic patients had been previously resuscitated according to Surviving Sepsis Campaign guidelines [2] including fluid challenge in all and vasopressors in 25 patients, mostly norepinephrine (Table 1). Source control was ongoing in all. In the cardiogenic patients, 24 were receiving inotropic support with dobutamine, milrinone, or levosimendan (Table 1). Only nine patients were under vasodilator therapy. Hospital mortality for the whole group was 22%.

No severe adverse events such as arrhythmias or cardiac arrest during intubation were registered. Thirty-three patients used vasopressors before intubation (Table 1), of whom 14 required a transitory increase in norepinephrine dose. Of the reminder 70 patients, 17 required one or two 8 mg ephedrine bolus plus an additional 250 ml normal saline bolus during the study protocol.

**Figure 1:**
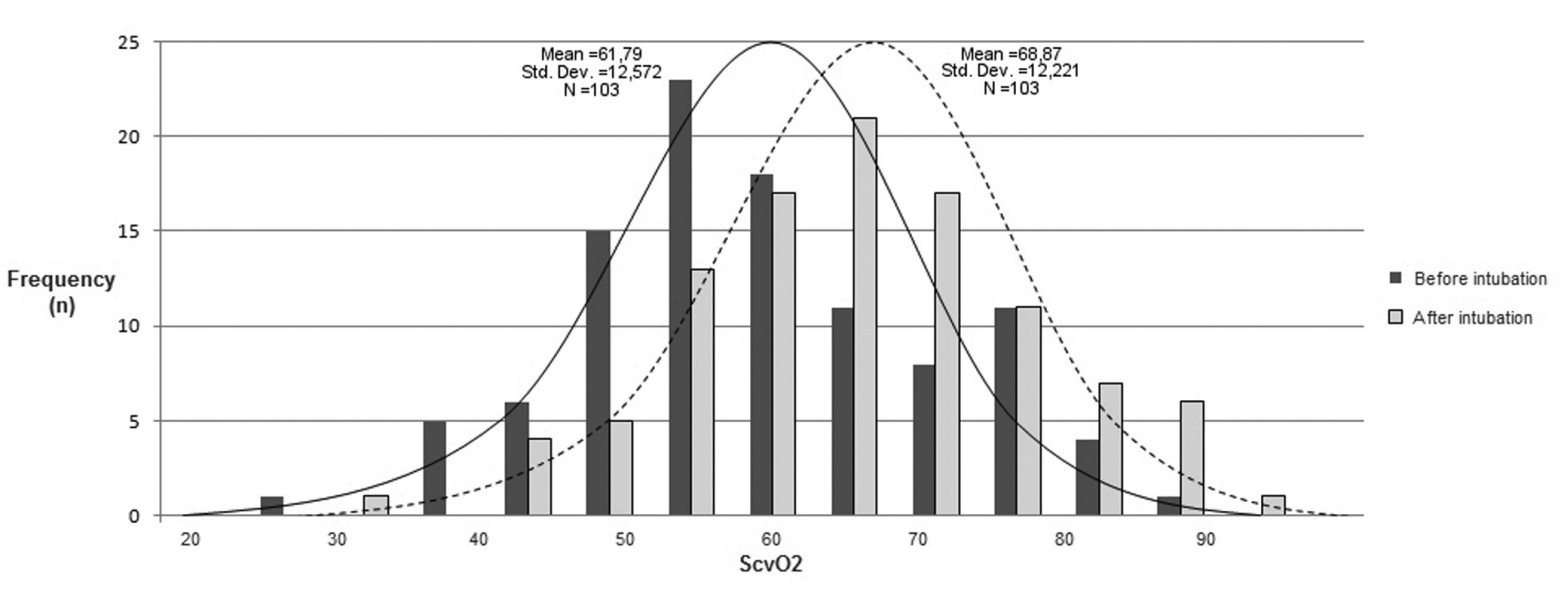
Distribution of central venous oxygen saturation before and after intubation. ScvO_2 _= central venous oxygen saturation.

**Figure 2:**
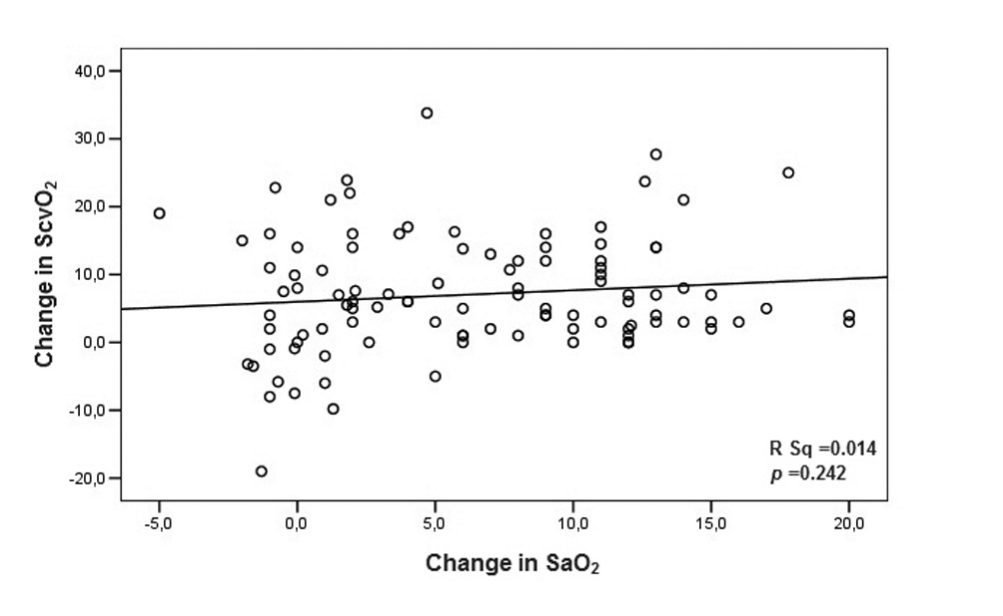
Correlation between changes in central venous oxygen saturation and arterial oxygen saturation after intubation. SaO_2 _= arterial oxygen saturation; ScvO_2 _= central venous oxygen saturation.

In the whole group, ScvO_2 _increased after intubation in 84 of 103 patients (81.6%) from 61.8 ± 12.6% to 68.9 ± 12.2% (*P *< 0.0001; Table 2 and Figure 1). ScvO_2 _increased also significantly in both septic and non-septic patients (Table 2). Changes in ScvO_2 _were independent from changes in SaO_2 _as demonstrated by a non-significant correlation between both (r^2 ^= 0.014, *P *= 0.242; Figure 2). As a whole, 78 (75.7%) patients were intubated with a ScvO_2 _less than 70% and 21 (26.9%) normalized the parameter after this sole intervention.

**Table 2:** Study variables before vs. after intubation

	Before intubation	After intubation	*P *value
SaO_2 _(%)	90.6 ± 7.5	97.0 ± 2.9	< 0.001
O_2_ER (%)	32.1 ± 10.8	29.2 ± 11.6	0.002
Heart rate (beats/min)	103.7 ± 25.2	96.4 ± 23.1	0.020
Respiratory rate (breaths/min)	29.1 ± 6.2	15.2 ± 3.1	< 0.001
MAP (mmHg)	67.8 ± 19.6	57.5 ± 21.1	< 0.001

MAP = mean arterial pressure; O_2_ER = oxygen extraction; SaO_2 _= arterial oxygen saturation.

*P *< 0.05 considered as significant.

We also explored the impact of the maneuver over ScvO_2 _according to pre-intubation values of ScvO_2 _and SaO_2_. We found a significant increase in ScvO_2 _in patients with baseline ScvO_2 _less than 70% independent of baseline SaO_2. _Only patients with ScvO_2 _more than 70% failed to increase the parameter after intubation (Table 3).

**Table 3:** Changes in ScvO2 after intubation for different subgroups

	ScvO_2 _(%)		
	Before intubation	After intubation	*P *value
All patients	61.8 ± 12.6	68.9 ± 12.2	< 0.001
Presence of severe sepsis			
Yes (n = 48)	63.6 ± 11.9	71.1 ± 12.0	< 0.001
No (n = 55)	59.3 ± 13.1	65.6 ± 11.6	< 0.001
According to baseline ScvO_2_			
< 70% (n = 76)	56 ± 8.4	64.8 ± 10.8	< 0.001
≥ 70% (n = 27)	78 ± 6.7	80.2 ± 8.1	0.181
According to baseline SaO_2_			
< 90% (n = 42)	54.1 ± 8.0	61.5 ± 11.4	< 0.001
≥ 90% (n = 61)	67.1 ± 12.5	73.0 ± 10.0	< 0.001

SaO_2 _= arterial oxygen saturation; ScvO_2 _= central venous oxygen saturation.

*P *< 0.05 considered as significant.

As a whole, oxygen extraction decreased in 56 patients (54.4%) by more than 2.5%, but increased more than 2.5% in 32 patients (31%) compared with baseline. As expected, patients who decreased O_2_ER after intubation, exhibited higher pre-intubation respiratory rates (30.7 ± 6.3 *vs*. 25.3 ± 4.0; *P *= 0.047). Mean arterial pressure (MAP), HR, and RR decreased also significantly after intubation (Table 2).

Septic and non-septic subgroups showed the same trends in physiologic variables after intubation, except for a higher decrease in O_2_ER in septic patients, and in MAP in the non-septic subgroup.

## Discussion

Our study demonstrates that emergency intubation markedly improves ScvO_2 _in both septic and non-septic patients. Changes in ScvO_2 _were consistent across the studied subgroups, regardless of the cause of intubation and baseline arterial oxygen saturation. In contrast, the effects on oxygen extraction were more variable. In almost 30% of the patients this sole maneuver increased ScvO_2 _over 70%, a level considered as a resuscitation goal by current guidelines [2].

The role of ScvO_2 _as a reliable marker of global dysoxia has been widely accepted [1,2]. Nevertheless, no study has replicated the very low ScvO_2 _values of the EGDT trial [1]. Low ScvO_2 _values are present in less than 21% of ICU patients with septic shock or respiratory failure [5,6]. Interestingly, the study by van Beest and colleagues 83% of patients were already intubated before the first ScvO_2 _sampling [6]. In fact, our low pre-intubation ScvO_2 _values in septic patients closely resemble baseline data from the EGDT trial [1], although ScvO_2 _values after intubation are quite similar to those previously reported in the ICU setting [5,6,9].

Is normalization of ScvO_2 _after intubation a reliable indicator of a successful resuscitation? Our data show that ScvO_2_, as expected, is highly sensitive to intubation. We believe that early normalization of this sole parameter after intubation should be interpreted with caution. Either an increase in SaO_2 _in some patients, or a decrease in cerebral and respiratory muscles VO_2_, may both increase ScvO_2_, but not necessarily reflect an improvement in global perfusion. In concordance, a recent study challenged the sensitivity of a ScvO_2 _more than 70% as a marker of an adequate DO_2_/VO_2 _balance after resuscitation in the ICU setting [10]. Therefore, we strongly believe that a multimodal approach including other parameters such as clinical perfusion, venous-arterial partial pressure of carbon dioxide gradient or lactate, must be used to assess perfusion, particularly after intubation.

Although the aim of our clinical observational study was to evaluate the specific impact of emergency intubation on ScvO_2 _and not to explore the determinants of this response, some physiologic considerations are important. Several studies have shown that sedation and connection to mechanical ventilation can decrease oxygen consumption in the brain and respiratory muscles, the principal determinants of VO_2 _in the territories drained by the superior vena cava [11-17]. Supporting this concept, and as expected, we found that patients with higher pre-intubation RR exhibited more pronounced decreases in O_2_ER after the maneuver. Conversely, DO_2 _can also be affected by emergency intubation and mechanical ventilation either by increases in SaO_2 _or changes in cardiac output. The increase in intra-thoracic pressure and decrease in sympathetic outflow induced by the maneuver favor a decrease in venous return, vasomotor tone, and cardiac output. Thus, sometimes divergent changes in DO_2 _and VO_2 _can be induced by emergency intubation and could probably explain the variable effect on oxygen extraction. Our results demonstrate that in the majority of patients subjected to emergency intubation, either septic or not, the predominant effect is to increase ScvO_2_, although this cannot be predicted *a priori *in individual cases. Therefore, an early measurement of ScvO_2 _after intubation may facilitate interpretation of further changes during ScvO_2_-guided resuscitation.

Our study has several limitations. To obtain a more comprehensive physiologic interpretation of ScvO_2 _changes, future studies should directly assess the effects of intubation on each of the determinants of ScvO_2_. Unfortunately, we did not measure cardiac output due to the extreme emergency context. In addition, it should be confirmed if these short-term effects persist over time and if early normalization of ScvO_2_after emergency intubation truly represents a correction of global hypoperfusion.

Our results should not be interpreted as a mandatory recommendation to intubate every patient presenting with low ScvO_2 _during resuscitation. Some patients present severe hemodynamic instability after the maneuver. Clinicians must be aware of the inherent risks associated with emergency intubation, which should be balanced against the potential benefit.

## Conclusions

ScvO_2 _increases significantly in response to emergency intubation in critically ill septic and non-septic patients, although it is not clear if this truly represents an improvement in global dysoxia. Our findings may contribute to explain the discrepancy between EGDT trial and ICU reports concerning the incidence of low ScvO_2 _values in heterogeneous critically ill patients. When interpreting ScvO_2 _during early resuscitation, it is crucial to consider whether the patient has been intubated.

## Key messages

• ScvO_2 _increased significantly in response to emergency intubation in critically ill septic and non-septic patients.

• Changes in ScvO_2 _were consistent across the studied subgroups, regardless of the cause of intubation and baseline SaO_2_.

• In almost 30% of the patients, this sole maneuver increased ScvO_2 _to levels considered as a resuscitation goal by some current guidelines.

## Abbreviations

ALI: acute lung injury; APACHE: Acute Physiology and Chronic Health Evaluation; ARDS: acute respiratory distress syndrome; DO_2_: oxygen transport; EGDT: early goal directed therapy; FiO_2_: fraction of inspired oxygen; HR: heart rate; ICU: intensive care unit; MAP: mean arterial pressure; O_2_ER: oxygen extraction ratio; PEEP: positive end expiratory pressure; RR: respiratory rate; SaO_2_: arterial oxygen saturation; ScvO_2_: central venous oxygen saturation; SOFA: Sequential Organ Failure Assessment; VO_2_: oxygen consumption.

## Competing interests

The authors declare that they have no competing interests.

## Authors' contributions

GH conceived the study, and participated in its design and coordination and helped to draft the manuscript. AB conceived the study, and participated in its design and coordination and helped to draft the manuscript. RC (Rodrigo Cornejo) conceived the study, and participated in its design and coordination and helped to draft the manuscript. RC (Ricardo Castro) conceived of the study, and participated in its design and coordination and helped to draft the manuscript. MR performed the statistical analysis. JR, HP, JLN, and IA recruited patients. All authors read and approved the final manuscript.
